# Differential Activation of Pro-Survival Pathways by NIX/BNIP3L: An Expression-Level-Dependent Mechanism Governing PC12 Cell Fate During H_2_O_2_-Induced Oxidative Stress

**DOI:** 10.3390/biology15110867

**Published:** 2026-05-31

**Authors:** Fanghui Ge, Jingxuan Shu, Ziqian Liu, Haixiang Ma, Minghong Cai, Xinyan Deng, Hong Zhang, Jiandong Wang

**Affiliations:** 1 Sichuan Provincial Engineering Laboratory for Prevention and Control Technology of Veterinary Drug Residue in Animal-Origin Food, School of Laboratory Medicine, Chengdu Medical College, Chengdu 610500, China; 202022038@cmc.edu.cn (F.G.); ziqianliu77@163.com (Z.L.); 13308012660@163.com (H.M.); 17828347540@163.com (X.D.); 1000360@cmc.edu.cn (H.Z.); 2 Experiment Teaching Demonstration Center of Laboratory Medicine, School of Laboratory Medicine, Chengdu Medical College, Chengdu 610500, China; 3 Department of Pediatrics, School of Clinical Medicine, Chengdu Medical College, Chengdu 610500, China; 18381084711@163.com; 4 School of Bioscience and Technology, Chengdu Medical College, Chengdu 610500, China; 15882474900@163.com; 5 Chengdu Medical College Office of Science and Technology, Chengdu 610500, China; 6 Key Laboratory of Nuclear and Radiation Damage Mechanisms and New Treatment Technology at Chengdu Medical College, Chengdu 610500, China

**Keywords:** NIX/BNIP3L, oxidative stress, mitophagy, apoptosis

## Abstract

Oxidative stress is a major contributor to neuronal apoptosis and subsequent neurofunctional deficits. This study investigates the dual role of the mitochondrial membrane-anchored protein NIX in PC12 cells, a model for mature neurons. We demonstrate that both overexpression and knockdown of NIX attenuate apoptosis under oxidative stress, albeit through distinct mechanisms. Overexpression of NIX promotes cell survival by activating NIX-mediated mitophagy, which clears damaged mitochondria and intracellular reactive oxygen species (ROS), thereby maintaining redox homeostasis. Conversely, knockdown of NIX reduces apoptosis primarily by diminishing the intrinsic pro-apoptotic function of the protein. Collectively, these findings reveal that NIX expression levels critically gate PC12 cell fate under oxidative stress by differentially activating pro-survival or anti-apoptotic pathways.

## Simple Summary

This study shows that the mitochondrial protein NIX has two different effects on neuron-like PC12 cells under oxidative stress. When NIX is overexpressed, it protects cells by clearing damaged mitochondria and reducing harmful reactive oxygen species (ROS) through a process called mitophagy. When NIX is knocked down, it also reduces cell death, but by lowering the protein’s natural pro-apoptotic activity. Together, these results indicate that the level of NIX expression determines whether cells survive or undergo apoptosis under oxidative stress.

## 1. Introduction

Oxidative stress serves as a common pathological basis for various neurodegenerative diseases and acute brain injuries [1]. It results from an imbalance between the formation of reactive oxygen species (ROS) and the impaired ability of an organism to detoxify these reactive intermediates or to repair the damage that they cause. Excessive ROS production originates primarily from mitochondrial electron transport chain leakage (at Complexes I and III) and NADPH oxidase (NOX) activation, particularly NOX2 in activated microglia during neuroinflammatory responses [2]. The subsequent oxidative damage manifests as lipid peroxidation of polyunsaturated fatty acids (PUFAs), protein carbonylation and nitration, as well as DNA oxidation (e.g., 8-hydroxy-2′-deoxyguanosine, 8-OHdG) and mitochondrial DNA damage [2,3]. Ultimately, these cascading events lead to neuronal apoptosis through both the intrinsic (mitochondrial) pathway—mediated by Bax/Bak activation, mitochondrial outer membrane permeabilization (MOMP), and cytochrome c release—and the extrinsic (death receptor) pathway involving caspase-8 activation [4], making oxidative stress a key driver of neuronal loss in neurological disorders.

A critical cellular response to oxidative damage is the selective removal of damaged mitochondria via mitophagy, a process essential for maintaining redox homeostasis [5]. The mitochondrial membrane protein NIX (also known as BNIP3L) serves as a key receptor for mitophagy [6,7]. However, NIX exhibits functional duality: beyond its pro-survival role in mitochondrial quality control, it can also engage apoptotic pathways through its transmembrane (TM) domain [8]. In chronic degenerative disorders such as Parkinson’s disease (PD) and Alzheimer’s disease (AD) [9,10], impaired function or reduced expression of NIX is often observed, leading to the accumulation of damaged mitochondria. In contrast, in acute brain injuries such as stroke [11,12] or ischemia-reperfusion [13,14], its expression is strongly induced, yet it may play a pro-apoptotic role. This positions NIX as a potential decision point for neuronal fate under stress, though its precise, context-dependent function remains unresolved.

Critically, prior studies have not systematically dissected how differing levels of NIX expression directly determine cell fate in a defined neuronal model of oxidative injury [15,16,17,18]. Although NIX has been recognized as a versatile regulator of cell fate [16,17], and its role in mediating mitophagy under various pathological conditions has been documented [7,9,11], whether its pro-survival or pro-apoptotic function predominates under specific conditions, or how these pathways are differentially activated, remains unknown.

To address this, our study employs a PC12 neuronal cell model subjected to H_2_O_2_-induced oxidative stress. We specifically investigate the expression-level-dependent role of NIX by implementing both gain-of-function (overexpression) and loss-of-function (knockdown) approaches. This design allows us to test the central hypothesis that NIX gates neuronal survival through distinct, expression-level-dependent mechanisms: at high levels, potentially promoting cytoprotective mitophagy, while at low levels, attenuating its intrinsic pro-apoptotic activity. Our findings aim to clarify this dual regulatory mechanism, providing a refined theoretical framework for targeting NIX in conditions associated with oxidative neuronal damage.

## 2. Materials and Methods

### 2.1. Cell Culture and Treatment

The rat adrenal pheochromocytoma PC12 cell line was obtained from the Cell Bank of Shanghai Institute of Biochemistry and Cell Biology (Shanghai, China). It is commonly employed to study the mechanism of action of nerve growth factor on cells, as well as the molecular regulatory mechanisms underlying neuronal development. The PC12 cells were cultured in DMEM medium containing 10% (*v*/*v*) fetal bovine serum (Biological Industries, Beit Haemek, Israel), 100 U/mL penicillin, and 0.1 mg/mL streptomycin (Gibco, Grand Island, NY, USA) in a 5% CO_2_ atmosphere at 37 °C. To establish in vitro cell models of over-expressing or silencing of NlX PC12 cells were infected with NlX lentivirus (Gene ID: NM 080888; Hanheng, Shanghai, China), NIX short hairpin RNA (shRNA) lentivirus (shRNA sequences: sense 5′-GGA AGA GTG GAG CCA TGA AGA TTC AAG AGATCT TCA TGG CTC CAC TCT TCC-3′ and antisense 5′-GGA AGA GTG GAG CCA TGA AGA TCT CTT GAATCT TCA TGG CTC CAC TCT TCC-3′; Hanheng, Shanghai, China) or corresponding control lentivirus respectively for 24 h and then treated with 10 ug/mL puromycin (Alomone, Jerusalem, Israel) for 14 days, according to the manufacturer’s protocol.

### 2.2. Viable Cell Counting

PC12 cells were seeded in six-well plates at a density of 3 × 10^5^ cells per well and cultured for 24 h. Cells were then collected into a centrifuge tube, the supernatant was discarded, and the cell pellet was resuspended in 1 mL of culture medium for subsequent use. An aliquot of 25 μL of the cell suspension was transferred to a new microcentrifuge tube containing 375 μL of phosphate-buffered saline (PBS) and mixed thoroughly. The prepared sample was then analyzed using a flow cytometer. Live cells were identified based on forward scatter (FSC) and side scatter (SSC) parameters, and the number of viable cells was calculated accordingly. At least 10,000 events were acquired per sample, and all experiments were independently repeated at least three times.

### 2.3. Detection of ROS

Intracellular ROS was detected with a Reactive Oxygen Species Assay Kit (Beyotime, Shanghai, China). In brief, PC12 cells were seeded into 6-well plates at a density of 3 × 10^5^ per well for 24 h. Then, the cells were treated with 10 µM DCFH-DA, a cell-permeable non-fluorescent probe useful for sensitive and rapid quantitation of oxygen-reactive species in response to oxidative metabolism for 20 min. The green fluorescence signal was detected by flow cytometric analysis (NovoExpress software, Version 1.6.2; Agilent Technologies, Santa Clara, CA, USA).

### 2.4. Measure Mitochondrial ROS with MitoSOX

MitoSOX is a mitochondria-targeted fluorescent probe that readily permeates the plasma membrane of live cells and selectively accumulates in mitochondria. Once inside the mitochondria, the probe is oxidized by superoxide and emits red fluorescence. The intracellular fluorescence intensity detected by fluorescence microscopy reflects mitochondrial reactive oxygen species (ROS) levels. Sterilized glass coverslips were placed in 24-well plates and washed three times with 500 µL D-hanks solution under gentle agitation, followed by a final rinse with 1 mL DMEM. Cells from each experimental group at the logarithmic growth phase were harvested, counted using flow cytometry, and resuspended at a density of 1 × 10^4^ cells per well in 1 mL complete medium. The cell suspensions were slowly added onto the coverslips and cultured at 37 °C in a humidified incubator containing 5% CO_2_. After 24 h, cells grown on coverslips were incubated with MitoSOX at a final concentration of 5 µM at 37 °C for 10 min in the dark. Cells were then gently washed three times with pre-warmed D-hanks solution, and images were captured using a fluorescence microscope.

### 2.5. Detection of H_2_O_2_

PC12 cells were seeded in six-well plates at a density of 3 × 10^5^ cells per well and cultured for 24 h. Cells were then collected into centrifuge tubes, and the supernatant was discarded. Hydrogen peroxide assay lysis buffer was added at a volume of 100–200 μL per 1 × 10^6^ cells, followed by thorough homogenization to disrupt and lyse the cells. The lysate was centrifuged at approximately 12,000× *g* for 3–5 min at 4 °C, and the supernatant was collected for subsequent analysis. A standard curve was generated using a standard solution. In a 96-well plate, 50 μL of each sample was mixed with 100 μL of hydrogen peroxide assay reagent and gently tapped to mix. After incubation for 30 min at room temperature (15–30 °C), the absorbance was measured at 560 nm. The hydrogen peroxide concentration in each sample was calculated based on the standard curve.

### 2.6. Apoptosis Assay

PC12 cells (1 × 10^5^) were cultured in a six-well plate and incubated at 37 °C for 24 h. Then, cells were treated with fresh culture medium containing 500 µM H_2_O_2_. After 12/24 h of incubation, using an Annexin V-FITC/propidium iodide (PI) apoptosis detection kit (Beyotime, Shanghai, China), cells were collected and stained, and then analyzed using flow cytometry.

### 2.7. Western Blot

The total proteins of the PCl2 cells were prepared using a RIPA Lysis Buffer (Beyotime, Shanghai, China). Protein samples were separated by 10% SDS-PAGE, and then transferred to 0.22-μm PVDF membranes (Millipore, Billerica, MA, USA). After blocking with 5% (*w*/*v*) non fat milk in TBS-T buffer (10 mM Tris-HCl, pH 7.5, 150 mM NaCl, 0.05% Tween 20) at 37 °C for 1 h, the membranes were incubated overnight at 4 °C with primary antibodies to β-actin (1:5000; Zen Bioscience, Chengdu, China), NIX (1:1000; Cell Signaling Technology, Danvers, MA, USA), BCL-2 (1:1000; ABclonal, Wuhan, China). BAX (1:1000; ABclonal, Wuhan, China), BCL-XL (1:1000; ABclonal, Wuhan, China), BAK (1:1000; ABclonal, Wuhan, China), Caspase3 (1:1000; ABclonal, Wuhan, China), SDHA (1:1000; Abways, Shanghai, China), TIMM23 (1:1000; Abways, Shanghai, China), IMMT (1:1000; Abways, Shanghai, China), TOMM20 (1:1000; Abways, Shanghai, China), VDAC (1:1000; Abways, Shanghai, China), MEK (1:1000; Abways, Shanghai, China), p-MEK (1:1000; Abways, Shanghai, China), P38 (1:1000; Abways, Shanghai, China), p-P38 (1:1000; Abways, Shanghai, China), ERK (1:1000; Abways, Shanghai, China), p-ERK (1:1000; Abways, Shanghai, China), JNK (1:1000; Abways, Shanghai, China), p-JNK (1:1000; Abways, Shanghai, China), P53 (1:1000; Abways, Shanghai, China), Acetyl-p53 (1:1000; Abways, Shanghai, China), AKT (1:1000; Abways, Shanghai, China), p-AKT (1:1000; Abways, Shanghai, China), p-PI3K (1:1000; Abways, Shanghai, China), LC3-I (1:1000; Abways, Shanghai, China), LC3-II (1:1000; Abways, Shanghai, China), P62 (1:1000; Abways, Shanghai, China), BNIP3 (1:1000; Abways, Shanghai, China), respectively. The next day, after washing away the first antibodies, the membranes were incubated with secondary antibodies (1:2000; Beyotime, Shanghai, China) at 37 °C for 1 h. Protein bands were detected by an ECL chemiluminescent detection kit (Millipore, Billerica, MA, USA). Data represent at least three independent experiments.

### 2.8. Detection of Cellular ATP Levels

ATP levels in cell lysates were measured using an Enhanced ATP Assay Kit (Cat. No. S0027, Beyotime, Shanghai, China). Briefly, cells were lysed on ice using the ATP lysis buffer provided with the kit. The lysates were then centrifuged at 12,000 rpm for 10 min at 4 °C, and the supernatant was collected for further analysis. The ATP detection reagent was diluted with the ATP detection reagent diluent to prepare the ATP detection working solution. In a 96-well plate, the sample or ATP standard was mixed with the ATP detection working solution and incubated for 10 min at room temperature in the dark. Luminescence intensity was measured using a chemiluminescence detection instrument. A standard curve was generated by plotting the luminescence values of ATP standard solutions against their corresponding ATP concentrations. The ATP concentration in each sample was calculated based on the standard curve. Meanwhile, an aliquot of the same cell lysate supernatant was taken to determine total protein concentration using a BCA protein assay kit. The results were normalized to protein content and expressed as nanomoles of ATP per milligram of protein (nmol/mg protein).

### 2.9. Mitochondrial Membrane Potential by the JC-1 Assay

To detect the mitochondrial membrane potential in this study, a JC-1 assay kit was used (Beyotime Biotechnology, Shanghai, China). Based on the manufacturer’s instructions, primary PC12 cells from each indicated group in 24-well plates were stained with a JC-1 staining solution at 37 °C for 20 min while protected from light. Then, each well in the plate was washed twice with 1× JC-1 staining buffer, and the fluorescence intensity was measured by flowcytometric analysis (Agilent, NovoExpress, Santa Clara, CA, USA). The red to green fluorescence ratio reflected changes in the mitochondrial membrane potential.

### 2.10. Mitochondrial Mass by Mito-Tracker Green Staining and Flow Cytometric Analysis

Mitochondrial mass was evaluated using Mito-Tracker Green (C1048, Beyotime Biotechnology, Shanghai, China). Cells were seeded in 6-well plates and subjected to the indicated treatments. After treatment, cells were incubated with Mito-Tracker Green working solution diluted in complete culture medium at 37 °C for 30 min in the dark, according to the manufacturer’s instructions. Following staining, cells were washed twice with PBS, harvested by trypsinization, and resuspended in PBS. Fluorescence signals were detected using a flow cytometer equipped with a 488-nm laser, and emission was collected in the FITC channel. At least 10,000 events were acquired per sample. Data were analyzed using FlowJo software 1.4.0, and mean fluorescence intensity (MFI) was calculated for quantitative comparison. All experiments were independently repeated at least three times.

### 2.11. Lysosomal Mass by Lyso-Tracker Red Staining and Flow Cytometric Analysis

Lysosomal mass is assessed using Lyso-Tracker Red (C1046, Beyotime Biotechnology, China). Cells were seeded in 6-well plates and subjected to the indicated treatments. After treatment, cells were incubated with Lyso-Tracker Red working solution diluted in complete culture medium at 37 °C for 30 min in the dark, according to the manufacturer’s instructions. Following incubation, cells were washed twice with PBS, harvested by trypsinization, and resuspended in PBS. Fluorescence was measured using a flow cytometer equipped with a 561-nm laser, and emission was collected in the PE channel. At least 10,000 events were recorded per sample. Data were analyzed using FlowJo software, and MFI was calculated for quantitative comparison. All experiments were independently repeated at least three times.

### 2.12. Reagents

The following reagents were used: DMEM medium (Gibco, Grand Island, NY, USA; Cat. No. 11965092); fetal bovine serum (Biological Industries, Beit Haemek, Israel; Cat. No. 26010074); penicillin–streptomycin mixture (Gibco, 15640055); puromycin (Alomone, Jerusalem, Israel); RIPA lysis buffer (Beyotime, Shanghai, China; Cat. No. P0013B); DCFH-DA reactive oxygen species assay kit (Beyotime, S1105S); MitoSOX red mitochondrial superoxide indicator (Thermo Fisher Scientific, Waltham, MA, USA; Cat. No. M36008); JC-1 mitochondrial membrane potential assay kit (Beyotime, C2003S); Mito-Tracker Green (Beyotime, C1048); Lyso-Tracker Red (Beyotime, C1046); Annexin V-FITC apoptosis detection kit (Beyotime, C1062S); hydrogen peroxide assay kit (Beyotime, S0038); ATP assay kit (Beyotime, S0027).

### 2.13. Correlation Analysis Between ARMCX3 and BNIP3L Expression Levels

Microarray datasets GSE104704 and GSE26927 were downloaded from the Gene Expression Omnibus (GEO) database, together with their corresponding platform annotation files. Probe IDs were converted into gene symbols according to the annotation information, and the expression data corresponding to ARMCX3 and BNIP3L were extracted for subsequent analysis. Normalized log_2_-transformed expression values were used for downstream analyses. All statistical analyses and data visualization were performed using the R software environment (version 4.3.3). Pearson’s correlation analysis was conducted in R to evaluate the association between ARMCX3 and BNIP3L expression levels, and the correlation coefficient (R), together with the corresponding two-tailed *p* value, was calculated. Correlation scatter plots and linear regression fitting curves were generated using the ggplot2 package.

### 2.14. Statistical Analysis

Data were expressed as the means ± SD. Statistical analysis was performed by using SPSS 19.0. The comparisons between two groups were performed with *t* test, while comparisons among three or more groups were performed using ANOVA with post hoc Tukey’s test to correct multiple comparisons. The statistical significance was defined as a *p* value < 0.05.

## 3. Results

### 3.1. NIX Knockdown Promotes PC12 Cell Survival and Inhibits Apoptosis

**Figure 1:**
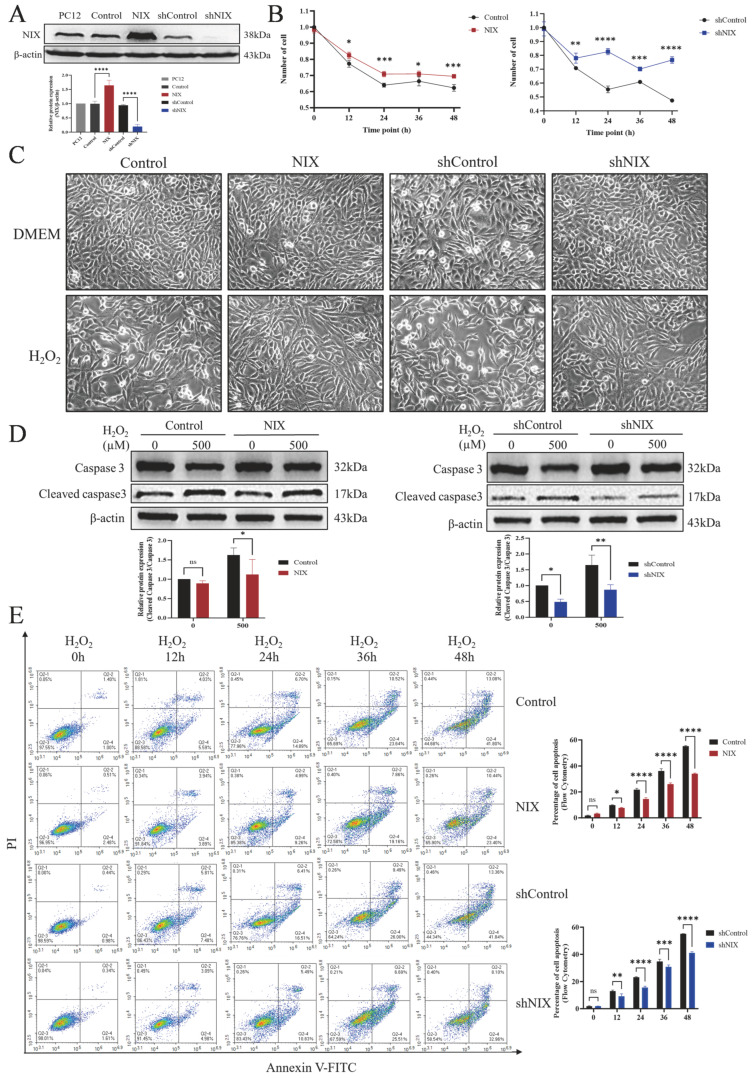
Both overexpression and knockdown of NIX attenuate H_2_O_2_-induced apoptosis in PC12 cells. (**A**) Western blot analysis confirming the establishment of NIX overexpression or knockdown models. (**B**) Cell viability after H_2_O_2_ treatment was evaluated using a live-cell counting assay. (**C**) Morphological changes in control PC12 cells and those with NIX overexpression or knockdown before and after treatment with 500 μM H_2_O_2_ after 24 h were observed under a light microscope. (**D**) Western blot analysis of caspase-3 and cleaved caspase-3 protein expression in control PC12 cells and those with NIX overexpression or knockdown before and after treatment with 500 μM H_2_O_2_ after 24 h. (**E**) Apoptotic rates were analyzed by flow cytometry. Data are presented as mean ± standard deviation (SD). ns, not significant; * *p* ≤ 0.05, ** *p* ≤ 0.01, *** *p* ≤ 0.001, **** *p* ≤ 0.0001. The uncropped/original scans of all protein gel images are available in the Supplementary Material.

To further investigate the regulatory role of NIX in oxidative stress of PC12 cells, we achieved the overexpression and knockdown of the NIX gene in PC12 cells via lentiviral transfection (Figure 1A). To verify the effect of NIX on the viability of PC12 cells under H_2_O_2_-induced oxidative stress, we detected the number of viable cells after treatment with 500 μM H_2_O_2_ (Figure 1B). The results showed that the cell viability of both NIX-overexpressing and NIX-knockdown PC12 cells was significantly higher than that of their respective control groups. The morphology and density of PC12 cells at 24 h post-H_2_O_2_ treatment were observed under an inverted microscope (Figure 1C), revealing that cells in the NIX-overexpressing and NIX-knockdown groups exhibited more intact morphology and significantly higher cell density. To clarify the regulatory effect of NIX on H_2_O_2_-induced apoptosis in PC12 cells, we determined the apoptotic level of cells by flow cytometry (Figure 1E). The results demonstrated that the apoptotic rate of NIX-overexpressing and NIX-knockdown PC12 cells was significantly lower than that of the control groups within 48h after H_2_O_2_ treatment. Meanwhile, the detection results of the apoptotic marker protein Caspase 3 and its activated, cleaved form also confirmed this conclusion (Figure 1D).

### 3.2. NIX Overexpression Alleviates Oxidative Stress Through Suppressing Intracellular ROS Accumulation

**Figure 2:**
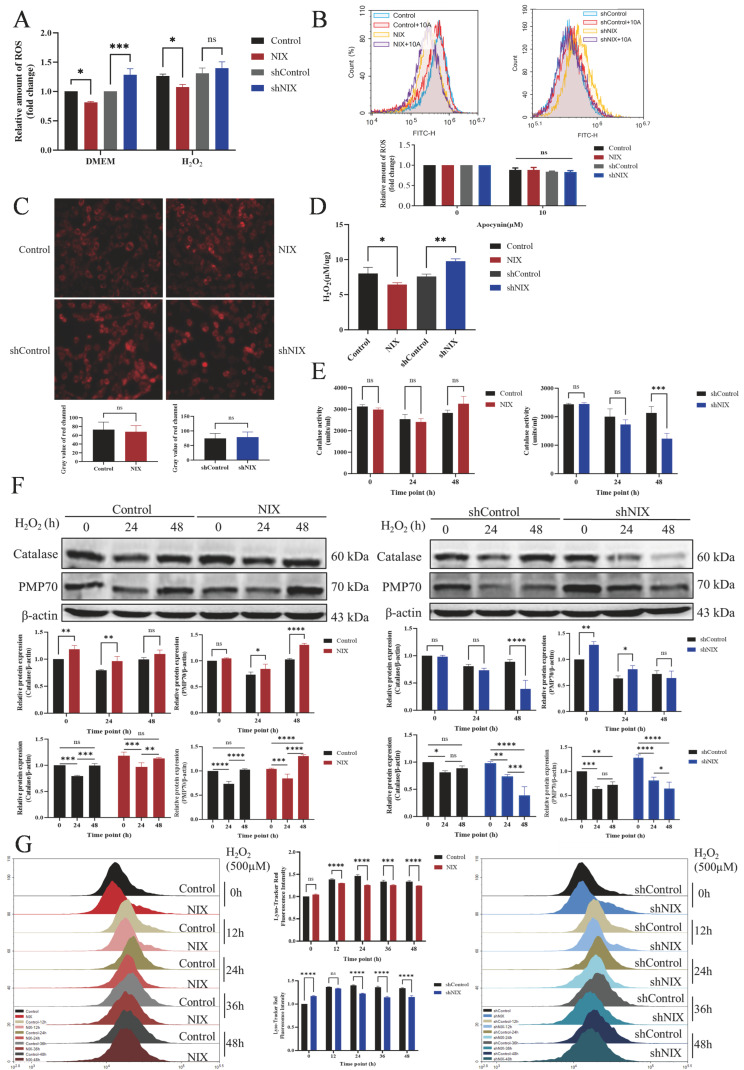
NIX regulates oxidative stress levels in PC12 cells under H_2_O_2_ stimulation. (**A**) Intracellular reactive oxygen species (ROS) levels in control PC12 cells and those with NIX overexpression or knockdown before and after treatment with 12 h of 500 μM H_2_O_2_. (**B**) ROS levels in control PC12 cells and those with NIX overexpression or knockdown after treatment with 10 μM apocynin. (**C**) Fluorescence intensity of intracellular mitochondrial superoxide. (**D**) Intracellular hydrogen peroxide levels. (**E**) Catalase activity assay. (**F**) Western blot analysis of Catalase and PMP70 protein expression in control PC12 cells and those with NIX overexpression or knockdown after 0, 24, and 48 h of 500 µM H_2_O_2_ treatment after 24 h. (**G**) Quantitative analysis of lysosomal mass in control PC12 cells and those with NIX overexpression or knockdown after 0, 12, 24, 36, and 48 h of 500 µM H_2_O_2_ treatment. Data are presented as mean ± standard deviation (SD). ns, not significant; * *p* ≤ 0.05, ** *p* ≤ 0.01, *** *p* ≤ 0.001, **** *p* ≤ 0.0001.

To explore the underlying molecular mechanism by which NIX regulates PC12 cell survival, we first detected the intracellular reactive oxygen species (ROS) level (Figure 2A). The results confirmed that H_2_O_2_ treatment significantly induced ROS accumulation in PC12 cells, while NIX overexpression effectively reduced ROS levels. After knocking down NIX expression, the level of ROS in the cells increased. NADPH oxidases (NOX) are key enzymatic sources of intracellular ROS, and Apocynin (4-hydroxy-3-methoxyacetophenone), a specific NOX inhibitor, can block NOX-mediated ROS production [19]. In this study, cells were treated with 10 μM Apocynin, and ROS changes were detected (S1A). The results showed that compared with their respective untreated groups, there was no significant decrease in ROS levels in either NIX-overexpressing or NIX-knockdown cells (Figure 2B), indicating that NOX is not the main source of ROS elevation in NIX-knockdown cells.

Mitochondrial ROS is mainly generated by electron leakage from the respiratory chain, with superoxide anion as its core form [20]. We used MitoSOX fluorescent probe to specifically detect mitochondrial superoxide anion levels, and the results showed no significant difference in fluorescence intensity between NIX-overexpressing/knockdown groups and the control group (Figure 2C), suggesting that ROS in NIX-knockdown cells is not mainly derived from mitochondrial superoxide anion. The above two results imply that ROS in NIX-knockdown cells may originate from mitochondrial hydrogen peroxide (H_2_O_2_). As a core member of the ROS family, H_2_O_2_ plays a critical hub role in the regulation of cellular oxidative stress [21]: it is mainly generated by the rapid dismutation of superoxide anion (O_2_^−^) leaked from the mitochondrial electron transport chain under the catalysis of superoxide dismutase (SOD), and can be converted into highly toxic hydroxyl radicals (·OH) through Fenton reaction/Haber-Weiss reaction in the presence of intracellular free iron/copper ions [3]. Further detection revealed that NIX overexpression significantly reduced intracellular H_2_O_2_ content, while early NIX knockdown led to massive accumulation of H_2_O_2_ in cells (Figure 2D). These results confirm that NIX overexpression can alleviate intracellular ROS accumulation by inhibiting the production of mitochondrial-derived H_2_O_2_.

In addition, we explored the intracellular ROS scavenging pathway to comprehensively clarify the molecular mechanism of ROS homeostasis disruption. Catalase and PMP70 (a major integral membrane protein on the peroxisomal membrane) are key components of the cellular antioxidant defense system. We detected intracellular Catalase activity (Figure 2E) and found no significant difference in enzyme activity between the NIX-overexpressing group and the control group. However, NIX-knockdown PC12 cells showed significantly reduced Catalase activity after 48 h of H_2_O_2_ treatment, indicating that NIX deficiency inhibits Catalase activity with the extension of H_2_O_2_ treatment time. The detection results of Catalase and PMP70 protein levels also verified this trend (Figure 2F), and we found that Catalase and PMP70 proteins exhibited dynamic changes under oxidative stress, while NIX deficiency led to regeneration disorders of these two proteins (Figure 2F). All the above results suggest that NIX plays an important role in maintaining the functional stability of peroxisomes.

Macroautophagy (referred to as autophagy) is an evolutionarily highly conserved lysosome-dependent degradation pathway and the main type of autophagy [22]. There is a sophisticated bidirectional regulatory network between autophagy and reactive oxygen species (ROS): on one hand, ROS act as key signaling molecules that induce autophagy through redox-sensitive pathways involving the regulation of autophagy-related (ATG) proteins and upstream signaling kinases [22]; on the other hand, autophagy serves as a cellular defense mechanism to clear ROS-generating damaged organelles and reduce oxidative stress [23]. Furthermore, the core machinery of macroautophagy provides the basis for selective autophagy pathways, including mitophagy, which specifically targets damaged mitochondria for degradation [23]. We used Lyso-Tracker Red fluorescent probe to detect the number of intracellular lysosomes (Figure 2G). The results showed that NIX-knockdown PC12 cells reversed the “high ROS-induced increase in lysosomal mass” under DMEM culture conditions, suggesting that NIX deficiency hinders the induction of macro-autophagy. Interestingly, the NIX-overexpressing group also showed a lower number of lysosomes, which on the one hand reflects less ROS accumulation in NIX-overexpressing cells, and on the other hand suggests that NIX overexpression may clear ROS through selective autophagy pathways (such as mitophagy). In summary, NIX overexpression can stabilize intracellular ROS homeostasis through a dual pathway of “inhibiting ROS production (especially mitochondrial-derived H_2_O_2_), maintaining the function of the ROS scavenging system”, thereby promoting the survival of PC12 cells under oxidative stress.

### 3.3. Overexpressed NIX Promotes Mitochondrial Homeostasis and Cell Survival Through Dimerization-Induced Mitophagy

**Figure 3:**
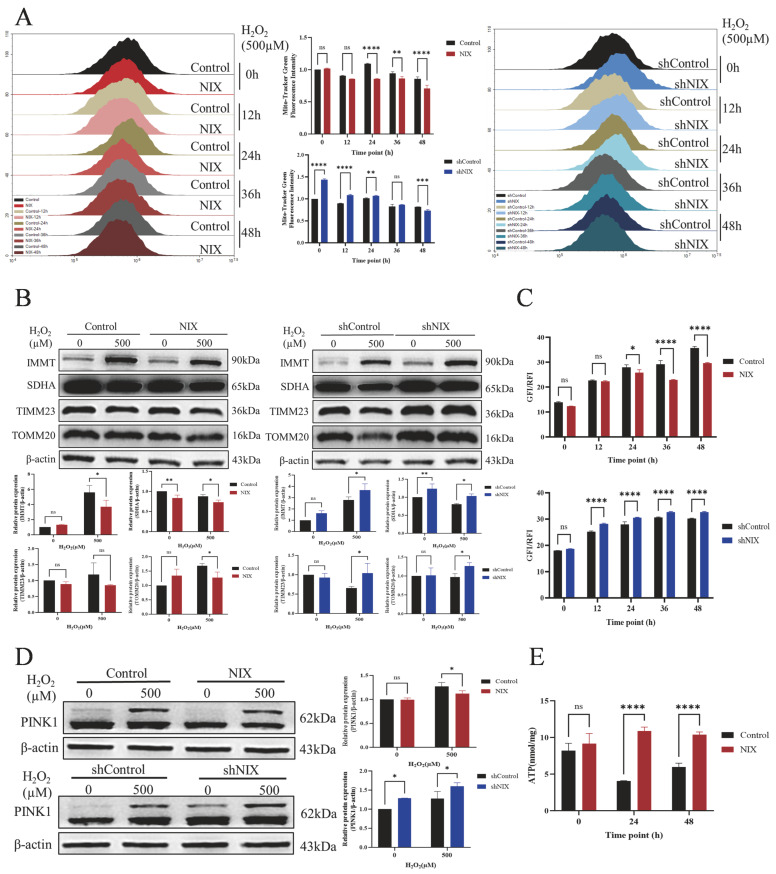
NIX affects mitochondrial abundance and homeostasis in PC12 cells under H_2_O_2_ stimulation. (**A**) Quantitative analysis of mitochondrial number in control PC12 cells and those with NIX overexpression or knockdown after 0, 12, 24, 36, and 48 h of 500 µM H_2_O_2_ treatment. (**B**) Western blot analysis of IMMT, SDHA, TIMM23, and TOMM20 protein expression in control PC12 cells and those with NIX overexpression or knockdown before and after treatment with 500 μM H_2_O_2_ after 24 h. (**C**) Mitochondrial membrane potential levels in control PC12 cells and those with NIX overexpression or knockdown after 0, 12, 24, 36, and 48 h of 500 µM H_2_O_2_ treatment. (**D**) Western blot analysis of PINK1 protein expression in control PC12 cells and those with NIX overexpression or knockdown before and after treatment with 500 μM H_2_O_2_ after 24 h. (**E**) Intracellular ATP levels. Data are presented as mean ± standard deviation (SD). ns, not significant; * *p* ≤ 0.05, ** *p* ≤ 0.01, *** *p* ≤ 0.001, **** *p* ≤ 0.0001.

To further confirm that NIX overexpression alleviates apoptosis induced by intracellular ROS accumulation primarily through mitophagy rather than simple macro-autophagy, we detected the number of mitochondria in viable cells using Mito-Tracker Green fluorescent probe. For the detection and assessment of autophagy, we referred to the study by Klionsky DJ et al. [24]. The results showed that under hydrogen peroxide induction, NIX-overexpressing PC12 cells exhibited a significantly reduced number of mitochondria. Interestingly, the phenomenon of massive mitochondrial accumulation in the early stage of NIX-knockdown PC12 cells was gradually alleviated with the extension of hydrogen peroxide treatment time (Figure 3A)—this change may be attributed to the failure of fluorescent staining caused by excessive mitochondrial damage at the late stage [25].

Additionally, we detected the expression levels of mitochondrial outer membrane protein TOMM20, inner membrane proteins TIMM and IMMT, as well as mitochondrial matrix protein SDHA (Figure 3B). Consistent with the results of mitochondrial fluorescence detection, the expression of mitochondrial membrane-related proteins in the NIX-overexpressing group was significantly downregulated after hydrogen peroxide treatment, while the opposite trend was observed in the NIX-knockdown group, suggesting that NIX overexpression mainly promotes the selective degradation of mitochondria by inducing mitophagy.

To further verify that NIX overexpression maintains mitochondrial function via mitophagy, we measured the mitochondrial membrane potential using JC-1 probe. The results indicated that NIX overexpression could effectively protect the stability of mitochondrial membrane potential under hydrogen peroxide induction, whereas the NIX-knockdown group showed a significant decrease in membrane potential (Figure 3C). PTEN-induced kinase 1(PINK1) is a key regulatory protein of mitophagy: when mitochondria are damaged, the loss of membrane potential impairs protein import, preventing PINK1 from entering the mitochondrial inner membrane and leading to its stable accumulation on the outer membrane [26]. Our detection revealed that after hydrogen peroxide treatment, the expression level of PINK1 in NIX-overexpressing PC12 cells was significantly reduced, while the opposite trend was observed in the NIX-knockdown group (Figure 3D).

As the energy factory of cells, the integrity of mitochondrial membrane structure is a prerequisite for adenosine triphosphate (ATP) synthesis. We further detected intracellular ATP content to reflect mitochondrial functional status. Consistent with the aforementioned results, NIX-overexpressing PC12 cells had significantly higher intracellular ATP content after hydrogen peroxide treatment (Figure 3E). Collectively, these results confirm that NIX overexpression maintains mitochondrial functional homeostasis by activating mitophagy, thereby alleviating oxidative stress-induced cell apoptosis.

**Figure 4:**
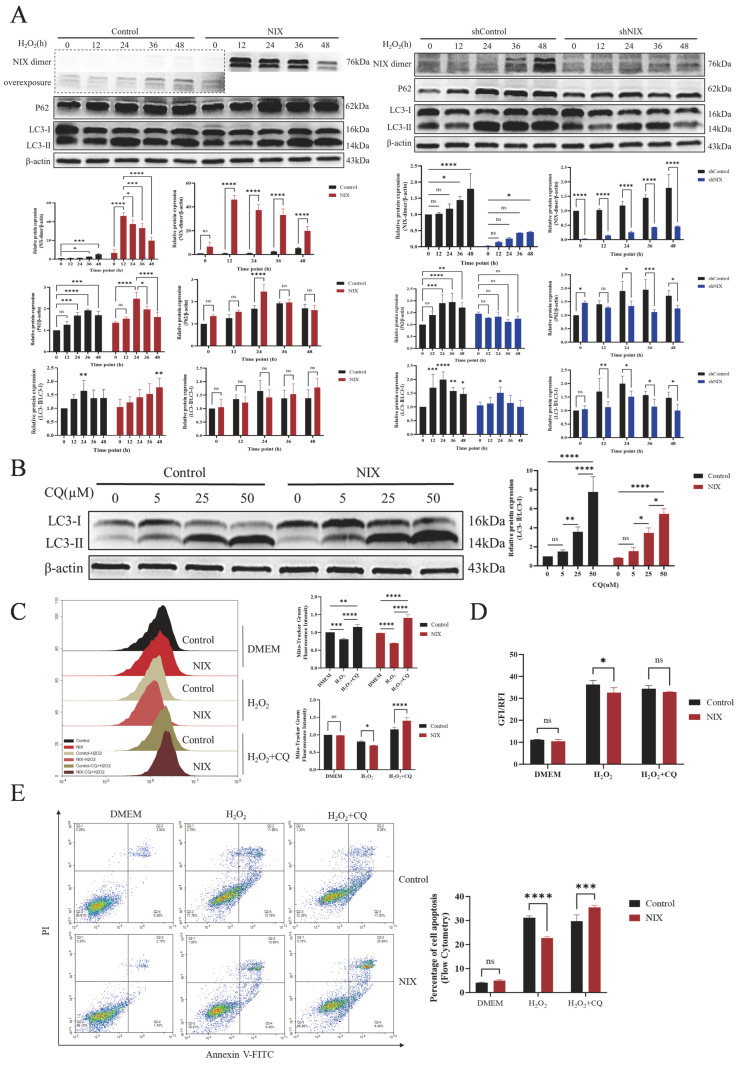
NIX regulates mitophagy in PC12 cells under H_2_O_2_ stimulation. (**A**) Western blot analysis of NIX dimers, p62, and LC3B protein expression in control PC12 cells and those with NIX overexpression or knockdown after 0, 12, 24, 36, and 48 h of 500 µM H_2_O_2_ treatment. (**B**) Western blot analysis of LC3B protein expression in control PC12 cells and those with NIX overexpression after 0, 24h of 0, 5, 25, 50 µM chloroquine (CQ) treatment. (**C**) Quantitative analysis of mitochondrial number in control PC12 cells and those with NIX overexpression treated with hydrogen peroxide alone or in combination with 0.25 µM CQ. (**D**) Mitochondrial membrane potential levels in control PC12 cells and those with NIX overexpression, treated with hydrogen peroxide alone or in combination with chloroquine. (**E**) Apoptotic rates in control PC12 cells and those with NIX overexpression after treatment with hydrogen peroxide alone or in combination with chloroquine, as analyzed by flow cytometry. Data are presented as mean ± standard deviation (SD). ns, not significant; * *p* ≤ 0.05, ** *p* ≤ 0.01, *** *p* ≤ 0.001, **** *p* ≤ 0.0001.

To further explore the specific molecular mechanism by which NIX overexpression mediates mitophagy, we detected the expression levels of autophagy-related proteins (P62, LC3B) and NIX dimers. The results showed that NIX knockdown significantly inhibited the formation of NIX dimers and blocked macro-autophagy, whereas NIX overexpression mainly induced mitophagy via promoting NIX dimerization at the early stage, rather than initiating macro-autophagy (Figure 4A).

To verify that NIX overexpression maintains mitochondrial function and cell survival through mitophagy, we used Chloroquine (CQ) to inhibit hydrogen peroxide-induced autophagic flux (Figure 4B). The results revealed that after CQ treatment, the original advantages of NIX-overexpressing group—“reduced mitochondrial number and stable mitochondrial membrane potential”—were reversed: the mitochondrial number increased significantly, and the stability of mitochondrial membrane potential was impaired (Figure 4C,D). More interestingly, following autophagy inhibition by CQ, the apoptotic rate of PC12 cells in the NIX-overexpressing group was significantly higher than that in the control group (Figure 4E). This suggests that NIX knockdown may block specific apoptotic pathways, thereby avoiding the exacerbation of apoptosis caused by the accumulation of damaged mitochondria after autophagy inhibition, while the protective effect of NIX overexpression on cells is completely dependent on the normal completion of mitophagy.

### 3.4. NIX Knockdown Exerts Anti-Apoptotic Effects by Directly Inhibiting the Apoptotic Signaling Pathway

We found that the apoptotic rate of NIX-overexpressing PC12 cells was significantly higher than that of the control group after autophagy inhibition by Chloroquine (CQ) (Figure 4D). Given that NIX possesses dual functions as a “pro-apoptotic factor” and a “mitophagic receptor”, we hypothesize that under the ideal condition where autophagy is completely inhibited by CQ, the pro-apoptotic function of NIX overexpression is prominent, which is highly consistent with our experimental results (Figure 4D). Conversely, NIX knockdown leads to the loss of both autophagic regulatory function and pro-apoptotic activity.

**Figure 5:**
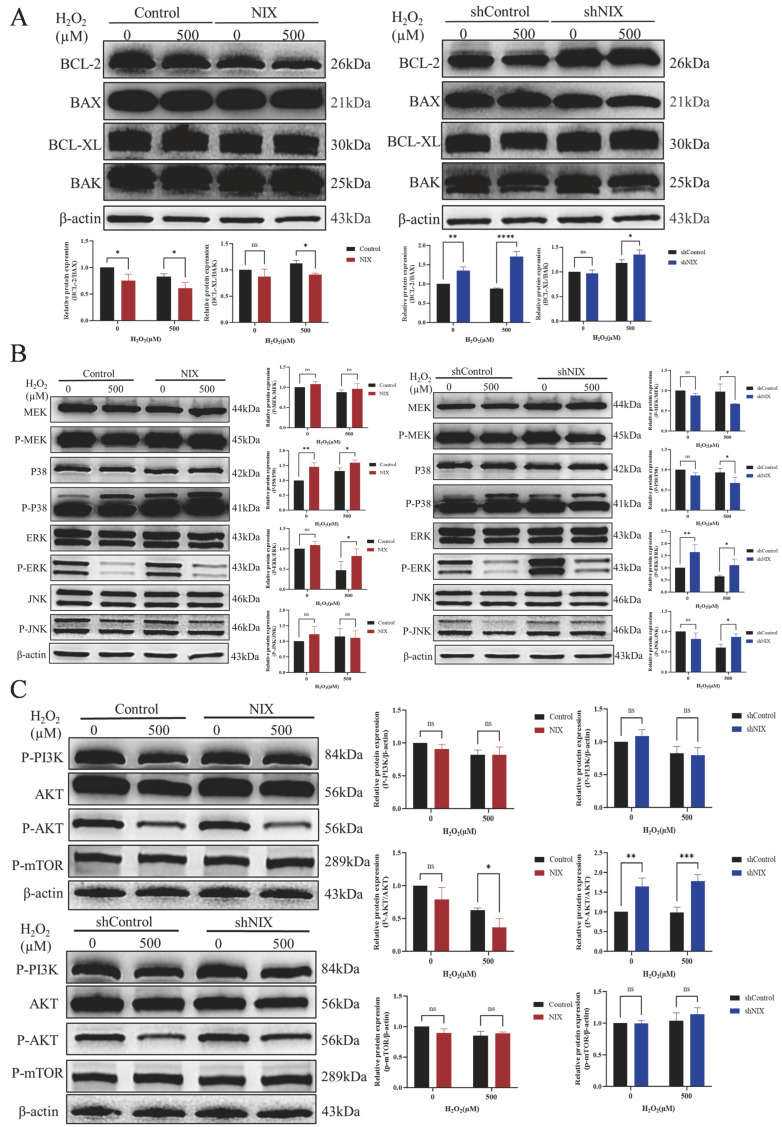
Changes in the expression of apoptosis-related proteins in PC12 cells under H_2_O_2_ stimulation. (**A**) Western blot analysis of Bcl-2, BAX, Bcl-XL, and BAK protein expression in control PC12 cells and those with NIX overexpression or knockdown before and after treatment with 500 μM H_2_O_2_ after 24 h. (**B**) Western blot analysis of core MAPK family members and their phosphorylated forms in control PC12 cells and those with NIX overexpression or knockdown before and after treatment with 500 μM H_2_O_2_ after 24 h. (**C**) Western blot analysis of phosphorylated proteins of key components in the PI3K–AKT–mTOR pathway in control PC12 cells and those with NIX overexpression or knockdown before and after treatment with 500 μM H_2_O_2_ after 24 h. Data are presented as mean ± standard deviation (SD). ns, not significant; * *p* ≤ 0.05, ** *p* ≤ 0.01, *** *p* ≤ 0.001, **** *p* ≤ 0.0001.

As NIX belongs to the BH3-only subfamily of the BCL protein family, we further detected the expression levels of anti-apoptotic proteins (Bcl-2, Bcl-XL) and pro-apoptotic proteins (BAK, BAX). The results showed that NIX knockdown directly increased the ratio of anti-apoptotic proteins to pro-apoptotic proteins, while NIX overexpression exhibited the opposite trend (Figure 5A). This result confirms that NIX knockdown can block the apoptotic pathway and maintain cell survival by directly regulating the balance of BCL family proteins.

To clarify the upstream molecular mechanism underlying the NIX knockdown-mediated anti-apoptotic phenotype (increased Bcl-2/BAX and Bcl-XL/BAK ratios), we detected the total protein expression and phosphorylation activation levels of core subtypes of the MAPK family (ERK1/2, p38 MAPK, JNK) under oxidative stress. The results revealed that NIX knockdown significantly altered the activation pattern of the MAPK pathway: the level of phosphorylated ERK1/2 (p-ERK1/2) was significantly increased, while the activation level of phosphorylated p38 MAPK (p-p38) was significantly downregulated (Figure 5B). There was no significant difference in the total protein expression of each subtype, indicating that NIX knockdown mainly affects the kinase activity of MAPK through regulating its phosphorylation modification, rather than altering protein synthesis and degradation.

Notably, following NIX knockdown, the level of phosphorylated JNK(p-JNK) was also increased under hydrogen peroxide stimulation, which contradicts the classic regulatory logic: as a core pro-apoptotic kinase under oxidative stress, JNK activation should initiate the mitochondrial apoptotic pathway by phosphorylating molecules such as Bim and Bcl-2, but this did not offset the anti-apoptotic phenotype mediated by NIX knockdown in our model.

Based on the antagonistic crosstalk between the JNK and PI3K-AKT-mTOR pathways [17], we hypothesize that NIX knockdown may induce a compensatory shift toward PI3K-AKT-mTOR signaling, thereby maintaining the anti-apoptotic state. Specifically, it is known that under certain stress conditions, activation of the PI3K/AKT pathway can negatively regulate the ASK1-JNK signaling cascade [27]. Furthermore, AKT has been reported to function as a critical connector in the crosstalk network, influencing cell survival and apoptosis by integrating signals from these two pathways [28]. In addition, the PI3K/AKT/mTOR signaling pathway is well recognized as a core regulator of cell survival and proliferation. Therefore, we propose that NIX knockdown leads to AKT activation, which in turn may suppress JNK-mediated pro-apoptotic signaling, thereby promoting cell survival under oxidative stress.

To verify this hypothesis, we further detected the phosphorylation expression levels of core molecules in the PI3K-AKT-mTOR pathway. The results showed that compared with the control group, the protein level of phosphorylated AKT (p-AKT, Ser473/Thr308 sites) and the p-AKT/AKT ratio were significantly increased in NIX-knockdown cells (Figure 5B); however, the expression levels of its upstream molecule phosphorylated PI3K (p-PI3K) and downstream molecule phosphorylated mTOR (p-mTOR) showed no significant changes (Figure 5B). This result indicates that the AKT activation induced by NIX knockdown is independent of the classic PI3K upstream regulatory pathway and is not further transmitted to the downstream mTOR kinase, representing a PI3K-independent and mTOR-uncoupled specific activation mode of AKT.

### 3.5. NIX Knockdown Antagonizes Apoptosis via Regulation of Mitochondrial Membrane Proteins

**Figure 6:**
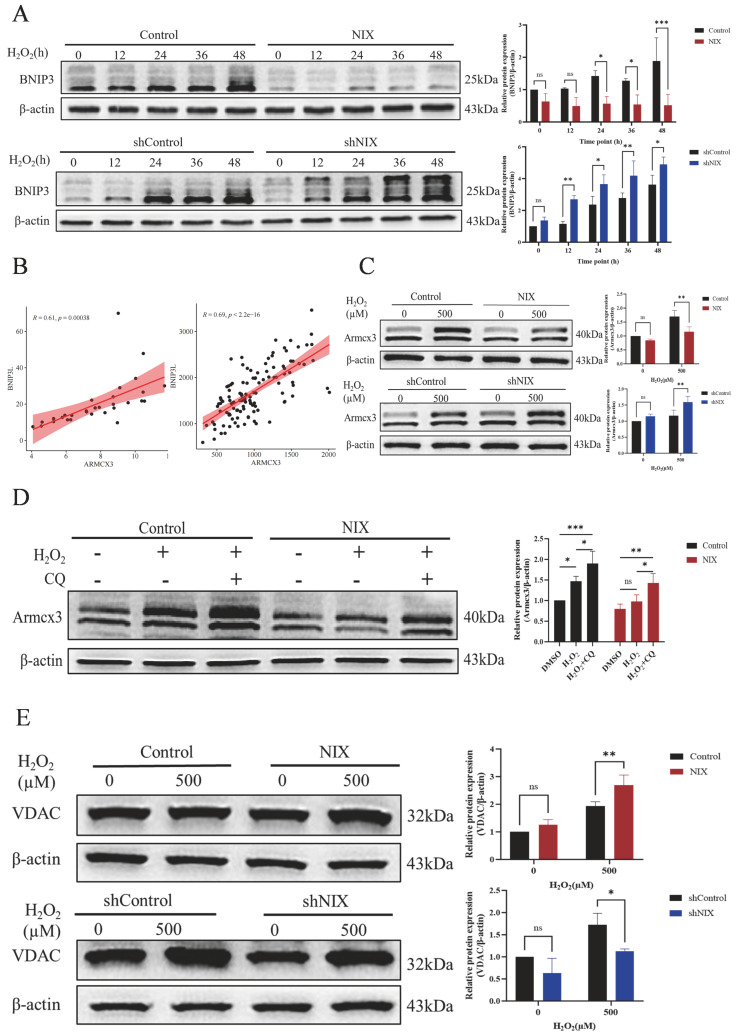
Correlation analysis between NIX/BNIP3L and ARMCX3. (**A**) Western blot analysis of BNIP3 protein expression in control PC12 cells and those with NIX overexpression or knockdown after 0, 12, 24, 36, and 48 h of 500 µM H_2_O_2_ treatment. (**B**) Correlation analysis between ARMCX3 and NIX/BNIP3L expression based on microarray datasets GSE104704 and GSE26927 from the GEO database. Scatter plots show gene expression levels across different samples along with linear regression fitting. Expression values are presented as normalized log_2_-transformed data. Pearson correlation analysis was performed, and the correlation coefficient (R) and corresponding *p* value are shown. The red regression line indicates the linear fit, and the shaded area represents the 95% confidence interval. (**C**) Western blot analysis of ARMCX3 protein expression in control PC12 cells and those with NIX overexpression or knockdown before and after treatment with 500 μM H_2_O_2_ after 24 h. (**D**) Western blot analysis of ARMCX3 protein expression in control PC12 cells and those with NIX overexpression treated with hydrogen peroxide alone or in combination with chloroquine. (**E**) Western blot analysis of VDAC protein expression in control PC12 cells and those with NIX overexpression or knockdown before and after treatment with 500 μM H_2_O_2_ after 24 h. Data are presented as mean ± standard deviation (SD). ns, not significant; * *p* ≤ 0.05, ** *p* ≤ 0.01, *** *p* ≤ 0.001.

As a protein localized to the outer mitochondrial membrane, NIX mediates mitophagy and apoptosis, which both directly act on mitochondria. BNIP3 and NIX belong to the BH3-only subfamily of the BCL protein family, sharing high structural homology. Both are localized to the outer mitochondrial membrane and exhibit similar pro-apoptotic and mitophagic functions. The results showed that BNIP3 was compensatorily upregulated in NIX-knockdown PC12 cells within 48 h of hydrogen peroxide treatment, while the opposite trend was observed in the NIX-overexpressing group (Figure 6A), which is consistent with the study by Humpton TJ et al. [29]. This suggests that the compensatory increase in BNIP3 cannot fully rescue the autophagic and apoptotic defects caused by NIX deficiency.

ARMCX3 (Armadillo repeat-containing X-linked protein 3) is a multifunctional scaffold protein localized to the outer mitochondrial membrane. Recent studies have demonstrated that ARMCX3 is a key molecular node integrating mitochondrial calcium (Ca^2+^) homeostasis, mitochondrial dynamic transport, and reactive oxygen species (ROS) signaling. It is highly expressed in the nervous system and plays important roles in multiple critical biological processes, including mitochondrial transport, neural development, tumor regulation, metabolic balance, and ROS signaling. By analyzing the GSE104704 and GSE26927 datasets, we found a significant positive correlation between the expression of ARMCX3 and BNIP3L (the gene encoding NIX), indicating a potential functional association between the two (Figure 6B). Similar to BNIP3, ARMCX3 protein was highly expressed in NIX-knockdown PC12 cells after hydrogen peroxide treatment, while the opposite result was observed in the NIX-overexpressing group (Figure 6C). We hypothesize that both NIX and ARMCX3 are induced under oxidative stress; in addition, NIX overexpression may directly clear damaged mitochondria or reduce ROS accumulation through mitophagy, thereby inhibiting ARMCX3 protein expression. To verify this hypothesis, we inhibited hydrogen peroxide-induced autophagy with Chloroquine and found that the ARMCX3 protein levels in both the NIX-overexpressing group and the control group were further increased compared with the group treated with hydrogen peroxide alone (Figure 6D), confirming that autophagy inhibition abrogates the regulatory effect of NIX on ARMCX3 expression.

VDAC (voltage-dependent anion channel) is a core component of endoplasmic reticulum-mitochondria contact sites (EMCS, also known as MAMs). It mainly regulates calcium signal transduction by forming the IP_3_R-Grp75-VDAC1 complex and participates in apoptotic signal transmission, serving as a key molecule for maintaining the structural integrity and functional homeostasis of EMCS [30]. A study by Jin MH et al. [31] confirmed that ARMCX3 can affect EMCS function by regulating mitochondrial distribution, maintaining mitochondrial Ca^2+^ levels and mitochondrial membrane potential stability, thereby inhibiting apoptosis. We detected the expression level of VDAC protein (Figure 6E), and the results showed that, unlike other mitochondrial membrane proteins (e.g., TOMM20, TIMM), the expression of VDAC protein in NIX-knockdown cells was significantly decreased after hydrogen peroxide treatment, while the opposite result was observed in the NIX-overexpressing group. These results suggest that NIX may regulate the expression of ARMCX3 to modulate the structure and function of endoplasmic reticulum-mitochondria contact sites (EMCS), ultimately governing the mitochondria-dependent apoptotic pathway.

## 4. Discussion

Neurons are particularly susceptible to oxidative damage due to their high oxygen consumption, rich content of polyunsaturated fatty acids (PUFAs), relatively weak antioxidant capacity, and high concentrations of transition metal ions (e.g., iron, copper) [32]. Under physiological conditions, low levels of reactive oxygen species (ROS) act as key signaling molecules to participate in the regulation of cell proliferation, differentiation, synaptic plasticity, and gene expression; however, excessive oxidative stress can lead to neuronal dysfunction and even death, which is a common pathological mechanism of various neurological diseases [33]. In this study, we established a state of severe oxidative stress in PC12 cells by hydrogen peroxide (H_2_O_2_) treatment, which mimics the state of neuronal oxidative stress induced by multiple pathological conditions such as neurodegenerative diseases, stroke, ischemia-reperfusion injury, inflammation, and ionizing radiation. Clinically, the acute phase of cerebral infarction mostly occurs 6–24 h after cerebral infarction, during which neurons exhibit edema, pallor, and obvious ischemic changes due to ischemia; the necrotic phase mainly occurs 24–48 h after cerebral infarction, during which a large number of neurons are shed, inflammatory cells infiltrate, and cerebral edema is significant [34,35]. Based on this clinical pathological feature, this study focused on the biological changes of PC12 cells within 48 h after H_2_O_2_ treatment. The results showed that NIX overexpression mainly eliminates damaged mitochondria through mediating mitophagy, reduces intracellular ROS accumulation, and thereby inhibits apoptosis; while NIX knockdown exerts an anti-apoptotic effect directly by blocking the apoptotic pathway. NIX possesses dual functions as a “pro-apoptotic factor” and a “mitophagic receptor”, and this characteristic provides PC12 cells with two distinct anti-apoptotic pathways under oxidative stress.

Mitophagy, a selective subtype of macro-autophagy, utilizes the core molecular mechanisms of macro-autophagy (e.g., Atg5/Atg7-dependent autophagosome formation) to recognize and degrade damaged or excess mitochondria via specific receptors (e.g., NIX). NIX mediates mitophagy primarily as a dimer, and dimerization is a prerequisite for its efficient recruitment of autophagy-related molecules [6]. Notably, NIX does not directly mediate non-selective macro-autophagy but initiates macro-autophagy through a specific mitophagic mechanism—specifically, activating autophagosome formation via two core pathways: the WIPI (WD repeat domain phosphoinositide-interacting protein)-ATG13 complex and the FIP200/ULK1 (unc-51 like autophagy activating kinase 1) complex [36], or disrupting the inhibitory interaction between Bcl-2 and Beclin1 [37], thereby enhancing the overall autophagic activity of cells. This explains the experimental phenomenon in our study: the macro-autophagy-related indicators (lysosomal mass, p62, and LC3B) in NIX-overexpressing cells were not higher than those in the control group, but NIX knockdown indeed inhibited macro-autophagy. Accumulating evidence has demonstrated that oxidative stress-induced organelle damage occurs in a spatiotemporal order [38,39], and mitochondria, as the primary site of ROS production, are the primary targets of oxidative stress [40]. Pathological levels of ROS preferentially attack components of the mitochondrial inner membrane, leading to the collapse of mitochondrial membrane potential and triggering a vicious cycle of “ROS-induced ROS release (RIRR)”, thereby amplifying the damage signal [3]. In the present study, NIX overexpression could directly mediate mitophagy via NIX dimers in the early stage, clear damaged mitochondria, and maintain the relative stability of intracellular ROS. Furthermore, we found that NIX dimerization is concentration-dependent on intracellular NIX levels, which is consistent with previous studies [6,41]. Meanwhile, dimeric NIX significantly increases the binding affinity to LC3/GABARAP family proteins, and initiates autophagosome biogenesis through the WIPI2-ATG13 pathway, mediating the tight attachment and expansion of the isolation membrane with mitochondria, and ultimately completing mitophagy [6].

NIX overexpression timely blocks intracellular ROS accumulation by initiating precise mitophagy in the early stage—at this point, NIX-mediated mitophagy dominates and inhibits its own pro-apoptotic activity; in contrast, NIX knockdown reduces both autophagic capacity and pro-apoptotic activity. As a BH3-only protein, NIX is intrinsically positioned to regulate apoptosis through its interaction with BCL-2 family members. Our results show that NIX knockdown increased the ratio of anti-apoptotic proteins (Bcl-2, Bcl-XL) to pro-apoptotic proteins (BAK, BAX), shifting the BCL-2 family balance toward survival and directly blocking the mitochondrial apoptotic pathway. To understand the upstream signals driving this shift, we examined the MAPK pathway, which comprises three core branches—ERK, JNK, and p38—that collectively regulate BCL-2 family protein expression through both direct phosphorylation and transcriptional control [42]. Specifically, NIX knockdown significantly increased phosphorylated ERK (p-ERK) while decreasing phosphorylated p38 (p-p38). Given that ERK activation upregulates Bcl-2 and Bcl-XL expression by activating transcription factors such as CREB and RSK, and directly phosphorylates Bcl-2 at Ser70 to enhance its stability [43,44,45], the observed increase in p-ERK provides a mechanistic explanation for the elevated anti-apoptotic protein levels in NIX-knockdown cells. Conversely, p38 and JNK typically promote apoptosis by mediating ubiquitin-dependent degradation of Bcl-2 and Bcl-XL, upregulating BAK/BAX expression via c-Jun and p53, and extending BAX half-life through Ser184 phosphorylation [46,47,48,49]. The decreased p-p38 in NIX-knockdown cells thus relieves this pro-apoptotic pressure, further contributing to the survival phenotype. Notably, p-JNK was elevated following NIX knockdown—a seemingly paradoxical finding given JNK’s established pro-apoptotic role. However, JNK can exhibit context-dependent functions, and its activation may reflect cross-talk with other survival pathways rather than driving apoptosis in this setting.

Given the known cross-talk between JNK and the PI3K-AKT-mTOR pathway, we further examined this axis. The PI3K-AKT-mTOR pathway is a central regulator of cell survival, growth, and metabolism [50]. Upon growth factor stimulation, PI3K generates PIP3, which recruits and activates AKT. Activated AKT then directly phosphorylates and inhibits pro-apoptotic proteins such as Bad and caspase-9, thereby promoting survival [51]. Our results showed that NIX knockdown increased phosphorylated AKT (p-AKT) at both Ser473 and Thr308 sites without altering p-PI3K or p-mTOR. This indicates that AKT activation occurs independently of the classic PI3K input and is not transmitted to downstream mTOR—a non-canonical, PI3K-independent and mTOR-uncoupled activation mode. This pattern of AKT activation may arise from stress-induced signals such as DNA damage (via DNA-PK) or elevated ROS, both of which can directly phosphorylate AKT. The functional consequence of this AKT activation is enhanced survival through direct inhibition of Bad and caspase-9, thereby reinforcing the anti-apoptotic phenotype already established by the rebalanced BCL-2 family and ERK-dominant MAPK signaling. Collectively, these findings reveal that NIX knockdown engages an interconnected multi-node survival network: it rebalances BCL-2 family proteins through ERK/p38 modulation, while simultaneously activating a non-canonical AKT survival pathway that operates independently of PI3K and mTOR. Together, these interconnected changes override the default apoptotic response and enable cell survival under severe oxidative stress.

Notably, NIX-knockdown cells exhibited enhanced resistance to apoptosis under oxidative stress. Although this phenotype favors short-term cell survival under damaging conditions, it may increase genomic instability if accompanied by persistent or unrepaired DNA damage, potentially promoting malignant transformation. Previous studies have shown that BNIP3 deficiency promotes cancer cell progression through increased glycolysis and angiogenesis [52]. As a homolog of BNIP3, downregulation of NIX may similarly affect cell fate through metabolic reprogramming. Furthermore, we found that in the NIX-knockdown group, expression levels of TOMM20, TIMM23, IMMT, and SDHA all increased after H_2_O_2_ treatment (Figure 3B), whereas VDAC expression significantly decreased (Figure 6E). This difference suggests that the regulatory mechanism of VDAC may differ from that of other mitochondrial membrane proteins. ARMCX3, as a mitochondrial outer membrane protein, showed an increasing trend (in NIX-knockdown cells) consistent with TOMM20 and others, but the distinct behavior of VDAC may be related to its specific localization and function at mitochondria-associated endoplasmic reticulum membranes (MAMs). The results of the present study indicate that NIX can regulate the protein level of ARMCX3 through mitophagy. It means that the function of ARMCX3 may be closely related to intracellular ROS homeostasis and mitophagy. However, the specific interaction mode between NIX and ARMCX3 (e.g., whether there is direct binding) and the molecular mechanism by which the two synergistically regulate mitochondrial function under disease conditions are key scientific issues that require in-depth investigation in future research.

In summary, this study elucidates the dual role of NIX in cellular response to oxidative stress. By establishing a model that mimics oxidative damage in neuronal cells, it was confirmed that elevated NIX expression primarily exerts a protective effect through initiating selective mitophagy, thereby effectively clearing damaged mitochondria. This process depends on the concentration-dependent dimerization of NIX via its transmembrane domain. In contrast, NIX knockdown, while impairing autophagic capacity, produces an anti-apoptotic effect by attenuating its inherent pro-apoptotic function.

## 5. Conclusions

In conclusion, this study demonstrates that NIX plays a dual role in determining PC12 cell fate under oxidative stress. Overexpression of NIX promotes concentration-dependent dimerization, initiating selective mitophagy that clears damaged mitochondria and reduces ROS accumulation. Conversely, NIX knockdown attenuates its intrinsic pro-apoptotic activity, which also suppresses apoptosis but at the cost of impaired autophagy. Collectively, NIX provides two distinct adaptive strategies—precise mitophagy or direct blockade of apoptosis—to counteract oxidative injury.

## Data Availability

The original contributions presented in this study are included in the article. Further inquiries can be directed to the corresponding author.

## Supplementary Materials

The following supporting information can be downloaded at: https://www.mdpi.com/article/10.3390/biology15110867/s1, Figure S1: Intracellular ROS levels after treatment with varying concentrations of apocynin are illustrated. Supplementary File S1: Original uncropped Western blot images.
