# Supplementary material for: Differential Activation of Pro-Survival Pathways by NIX/BNIP3L: An Expression-Level-Dependent Mechanism Governing PC12 Cell Fate During H2O2-Induced Oxidative Stress
Source: Biology (Basel). 2026 May 31;15(11):867. doi: 10.3390/biology15110867 (PMC13255857; doi:10.3390/biology15110867)
Supplement: Supplementary file 1 [file biology-15-00867-s001.zip › Figure S1.pdf]

Supplementary Materials:

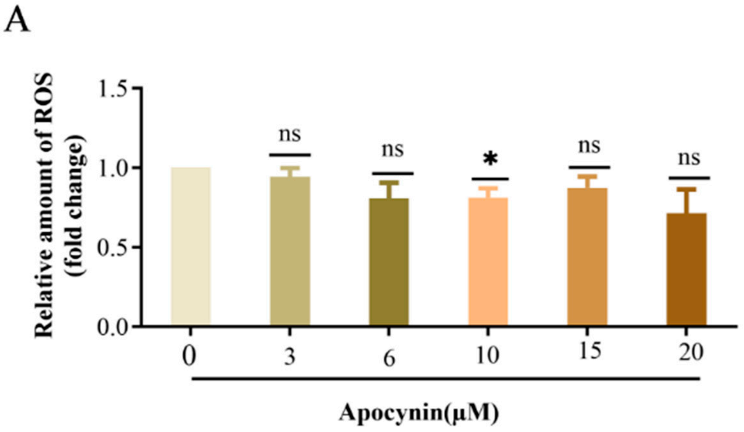

Supplementary Fig.1 Intracellular ROS levels after treatment with varying concentrations of apocynin are illustrated. Compared with the blank control group, the most pronounced decrease in ROS levels was observed at 10 μM Apocynin, whereas ROS levels began to increase at 15 μM, indicating that Apocynin exerts an oxidative effect on cells at higher concentrations. Based on these results, 10μM was identified as the optimal concentration.
